# Improved gut microbiota features after the resolution of SARS‑CoV‑2 infection

**DOI:** 10.1186/s13099-021-00459-9

**Published:** 2021-10-16

**Authors:** Flavio De Maio, Gianluca Ianiro, Gaetano Coppola, Francesco Santopaolo, Valeria Abbate, Delia Mercedes Bianco, Fabio Del Zompo, Giuseppe De Matteis, Massimo Leo, Antonio Nesci, Alberto Nicoletti, Maurizio Pompili, Giovanni Cammarota, Brunella Posteraro, Maurizio Sanguinetti, Antonio Gasbarrini, Francesca Romana Ponziani

**Affiliations:** 1 Dipartimento di Scienze di Laboratorio e Infettivologiche, Fondazione Policlinico Universitario Agostino Gemelli IRCCS, Rome, Italy; 2 Dipartimento di Scienze Biotecnologiche di Base, Cliniche Intensivologiche e Perioperatorie, Università Cattolica del Sacro Cuore, Rome, Italy; 3 Dipartimento di Medicina e Chirurgia Traslazionale, Università Cattolica del Sacro Cuore, Rome, Italy; 4 CEMAD Digestive Disease Center, Fondazione Policlinico Universitario Agostino Gemelli IRCCS, Rome, Italy; 5 Internal Medicine, Fondazione Policlinico Universitario Agostino Gemelli IRCCS, Rome, Italy; 6 Dipartimento di Scienze Mediche e Chirurgiche, Fondazione Policlinico Universitario A. Gemelli IRCCS, Rome, Italy; 7 Division of Internal Medicine and Gastroenterology, Hepatology Unit, Fondazione Policlinico Universitario Agostino Gemelli IRCCS, Università Cattolica del Sacro Cuore, Rome, Italy

**Keywords:** Gut microbiota, SARS-CoV-2, COVID-19, Pneumonia

## Abstract

**Background:**

The severe acute respiratory syndrome coronavirus 2 (SARS‑CoV‑2) has a tropism for the gastrointestinal tract and several studies have shown an alteration of the gut microbiota in hospitalized infected patients. However, long-term data on microbiota changes after recovery are lacking.

**Methods:**

We enrolled 30 patients hospitalized for SARS‑CoV‑2-related pneumonia. Their gut microbiota was analyzed within 48 h from the admission and compared with (1) that of other patients admitted for suspected bacterial pneumonia (control group) (2) that obtained from the same subject 6 months after nasopharyngeal swab negativization.

**Results:**

Gut microbiota alpha-diversity increased 6 months after the resolution of SARS-CoV-2 infection. Bacteroidetes relative abundance was higher (≈ 36.8%) in patients with SARS-CoV-2, and declined to 18.7% when SARS-CoV-2 infection resolved (p  =  0.004). Conversely, Firmicutes were prevalent (≈ 75%) in controls and in samples collected after SARS-CoV-2 infection resolution (p  =  0.001). Ruminococcaceae, Lachnospiraceae and *Blautia* increased after SARS-CoV-2 infection resolution, rebalancing the gut microbiota composition.

**Conclusion:**

SARS-CoV-2 infection is associated with changes in the gut microbiome, which tend to be reversed in long-term period.

**Supplementary Information:**

The online version contains supplementary material available at 10.1186/s13099-021-00459-9.

## Background

Several studies to date have analyzed the gut microbiota of patients with severe acute respiratory syndrome coronavirus 2 (SARS‑CoV‑2) infection both during and after disease resolution [1, 2]. However, information on long-term follow-up is lacking. Therefore, we evaluated changes to the gut microbiome six months after SARS-CoV-2 infection resolution in 30 Italian patients hospitalized for pneumonia in our center during the first wave of the pandemic.

## Study design and results

**Fig. 1:**
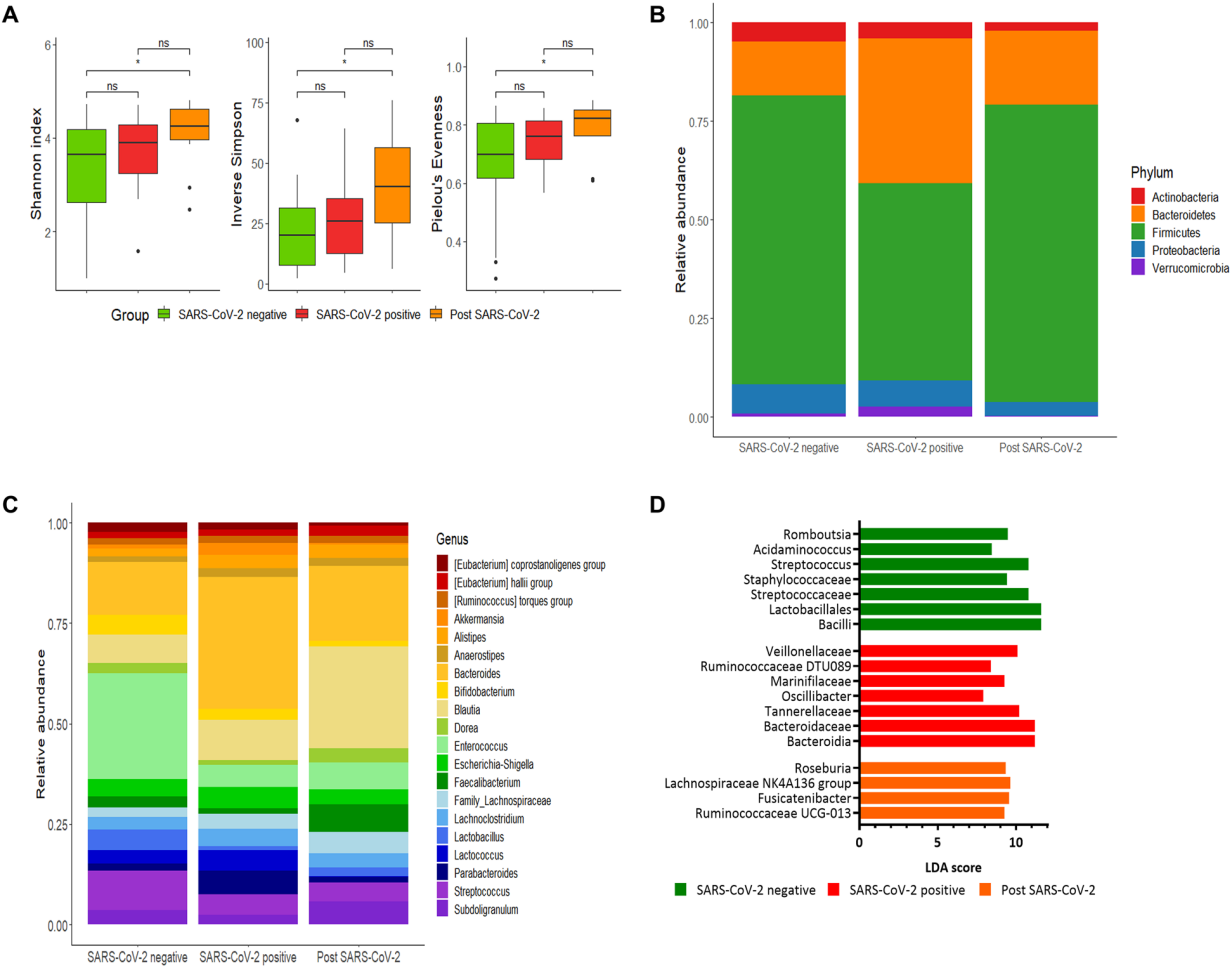
Gut microbiota analysis of patients with or without severe acute respiratory syndrome coronavirus 2 (SARS‑CoV‑2) infection. **A** alpha diversity measures; **B**, **C** phyla and genera distribution; **D** linear discriminant analysis (LDA) effect size (LEfSe) highlighting differently abundant taxa between the study groups

Faecal samples of 31 SARS-CoV-2-positive patients were harvested within 48 h from admission and prior to transfer to the intensive care unit (ICU), in order to minimize any impact of the pharmaceutical treatment on the gut microbiome, and again six months after discharge. During this , patients were regularly interviewed and none of them reported any infection/antibiotic treatment or symptoms of new onset. Eighteen patients hospitalized in the same period for SARS-CoV-2-unerlated pneumonia served as control group. Following hospital admission, at the time of fecal specimen collection, both control group (94.7%) and patients showing SARS-CoV-2 related pneumonia (71%) were receiving the same antibiotic treatment schedule, as per Hospital protocol. Faecal samples analysis was conducted as described in the Additional file 4. Characteristics of the study population are shown in Table 1. Gut microbiota alpha-diversity was similar between patients affected by either SARS-CoV-2 or non-SARS-CoV-2 pneumonia; it showed a slight enhanced trend after SARS-CoV-2 negativization, increasing significantly after the resolution of SARS-CoV-2 infection when compared with the control group (p  =  0.346, p  =  0.043 and p  =  0.048, Kruskal–Wallis Test, for Shannon index, inverse Simpson index and Pielou’s Evenness, respectively). In particular, equitability among bacterial species increased and appeared to be driven by microbial changes ensuing SARS-CoV-2 infection resolution (Fig. 1A). The PCoA of inter-individual variation based on weighted UniFrac distance showed a slender split among the study groups, although it was not statistically significant (p  =  0.114, PERMANOVA; Additional file 1: Figure S1). As shown in Fig. 1B, Bacteroidetes relative abundance was higher (≈ 36.8%) in SARS-CoV-2 positive patients than in those with SARS-CoV-2 negative pneumonia (≈13.6%), and declined to 18.7% when SARS-CoV-2 infection resolved (p  =  0.004, Kruskal–Wallis Test). Conversely, Firmicutes were prevalent (≈ 75%) in controls and in samples collected after SARS-CoV-2 infection resolution, while the relative abundance was lower (≈ 50%) in SARS-CoV-2 positive pneumonia group (p  =  0.001, Kruskal–Wallis Test).

**Table 1:** Clinical and demographic features of patients included in the study

	SARS-CoV2 + (31)	SARS-CoV2 − (18)	p value
Age	66.7 ± 14.4	67.1 ± 17.5	0.372
Sex			
Males	23 (74.2%)	11 (61.1%)	0.357
Females	8 (25.8%)	7 (38.9%)	
Gastrointestinal symptoms	13 (41.9%)	5 (27.8%)	0.249
Respiratory failure*	22 (73.3%)	6 (33.3%)	0.012
ARDS categories^a^			
III	14 (63.6%)	5 (83.3%)	
II	7 (31.8%)	1 (16.7%)	
I	1 (4.5%)	0	
History of chronic lung disease	4 (12.9%)	3 (16.7%)	0.512
Treatment during hospitalization			
Antibiotics	22 (71%)	18 (94.7%)	0.05
Antivirals	27 (90%)	3 (16.7%)	0.00
Hydroxychloroquine	29 (93.5%)	2 (10.5%)	0.017
Anti-IL-6 receptor monoclonal antibodies	8 (25.8%)	0	

Numeric variables are reported as mean  ±  standard deviation, categorical ones as frequencies and percentages

*Number of patients with respiratory failure during hospitalization (PaO_2_/FiO_2_ ratio  <  300)

Significance was evaluated by Wilcoxon signed rank test and chi-square test (IBM SPSS Statistics)

*SARS‑CoV‑2* severe acute respiratory syndrome coronavirus 2; *IL* interleukin

^a^III PaO_2_/FiO_2_ ratio 200–300, II PaO2/FiO2 ratio 100–200, I PaO2/FiO2 ratio  <  100

Focusing on the twenty most represented bacterial genera, we observed specific patterns of *Bacteroides*, *Blautia* and *Enterococcus* variations among groups (Fig. 1C). Particularly, *Bacteroides* and *Enterococcus* genera appeared to correlate inversely, with the former genus being increased in SARS-CoV-2 positive patients compared to the other groups (p  =  0.003), whereas the latter genus showed a decreasing trend (p  =  0.082). Finally, *Blautia* increased after SARS-CoV-2 infection resolution, rebalancing the gut microbiota composition (p  =  0.029). As expected, linear discriminant analysis (LDA) showed that bacterial elements belonging to Bacteroidetes (i. e., *Oscillibacter,* Ruminococcaceae DTU089*,* Bacteroidaceae*,* Bacteroidia*, Parabacteroides* and Tannerellaceae) were enriched in SARS-CoV-2 positive patients, whereas Lactobacillales, *Streptococcus, Staphylococcus and Acidoaminococcus* were increased in those with SARS-CoV-2-unrelated pneumonia (p  <  0.05, Kruskal–Wallis Test; Fig. 1D). Conversely, Lachnospiraceae (i. e., NK4A136 group, *Fusicantibacter* and *Roseburia*) and Ruminococcaceae UCG-013 were increased after SARS-CoV-2 infection resolution. DESeq2 analysis reported substantially the same results (Additional file 2: Table S1, Additional file 3: Table S2).

## Discussion

Our study conducted in a Western population confirms that gut microbiota alpha diversity is similar in patients with SARS-CoV-2 related or unrelated pneumonia. Nevertheless, we observed an increase in alpha-diversity after the resolution of SARS-CoV-2 acute infection. This may be related to the effects of SARS-CoV-2 on the gut microbiota, but the contribution of drugs (e.g., antibiotics) administered before hospitalization or during its initial phase may not be negligible. Nevertheless, alpha-diversity modification may be not sufficient to assess recovery of the healthy status.

Previous Chinese studies [3–5] compared the gut microbiota of patients with SARS-CoV-2-related and community-acquired pneumonia, confirming a surge of opportunistic pathogens and a reduction in commensals elements. Our data confirm Bacteroidetes enrichment in patients with COVID-19 [3]. Conversely, Firmicutes appeared to decline, whereas no significant variation was observed for Actinobacteria [3]. During SARS-CoV-2 infection, inflammation is a key determinant of disease severity [6–8]. Interestingly, cytokine storm is positively associated with *Bacteroides* relative abundance [3]. Conversely, *Blautia* with its anti-inflammatory properties may play an important role in the recovery from COVID-19 [9]. Indeed, interleukin-10 (IL-10) serum levels significantly decrease with the reduction of *Ruminococcus obeum* (otherwise *Blautia*) abundance [3], and this correlates with the failure in controlling host’s inflammatory response. This study suffers of the limitations of the small sample size, but our results corroborate those achieved on populations with different ethnicity. Even though many factors can affect the gut microbiota, our findings support the hypothesis of a specific impact of SARS-CoV-2 infection on the gut microbiota.

## Conclusions

This study supports the previous evidence that SARS-CoV-2 infection is associated with changes in the gut microbiome. However, many gut microbiome-related factors could influence the course of COVID-19, calling for more studies in the next future.

## Supplementary Information

**Additional file 1. **Principal coordinate analysis (PCoA) on weighted UniFrac distance of the gut microbiome of patient with severe acute respiratory syndrome coronavirus 2 (SARS‑CoV‑2)-related pneumonia, who were tested before and after resolution of the infection, and patients with SARS-CoV-2 unrelated pneumonia.

**Additional file 2. **DESeq2 analysis.

**Additional file 3. **DESeq2 analysis.

**Additional file 4. **Supplementary methods.

## Data Availability

Available upon reasonable request.
