# Supplementary material for: Improved gut microbiota features after the resolution of SARS‑CoV‑2 infection
Source: Gut Pathog. 2021 Oct 16;13:62. doi: 10.1186/s13099-021-00459-9 (PMC8520333; doi:10.1186/s13099-021-00459-9)

**Supplementary Figure 1:** Principal coordinate analysis (PCoA) on weighted UniFrac distance of the gut microbiome of patient with severe acute respiratory syndrome coronavirus 2 (SARS‑CoV‑2)-related pneumonia, who were tested before and after resolution of the infection, and patients with SARS-CoV-2 unrelated pneumonia.


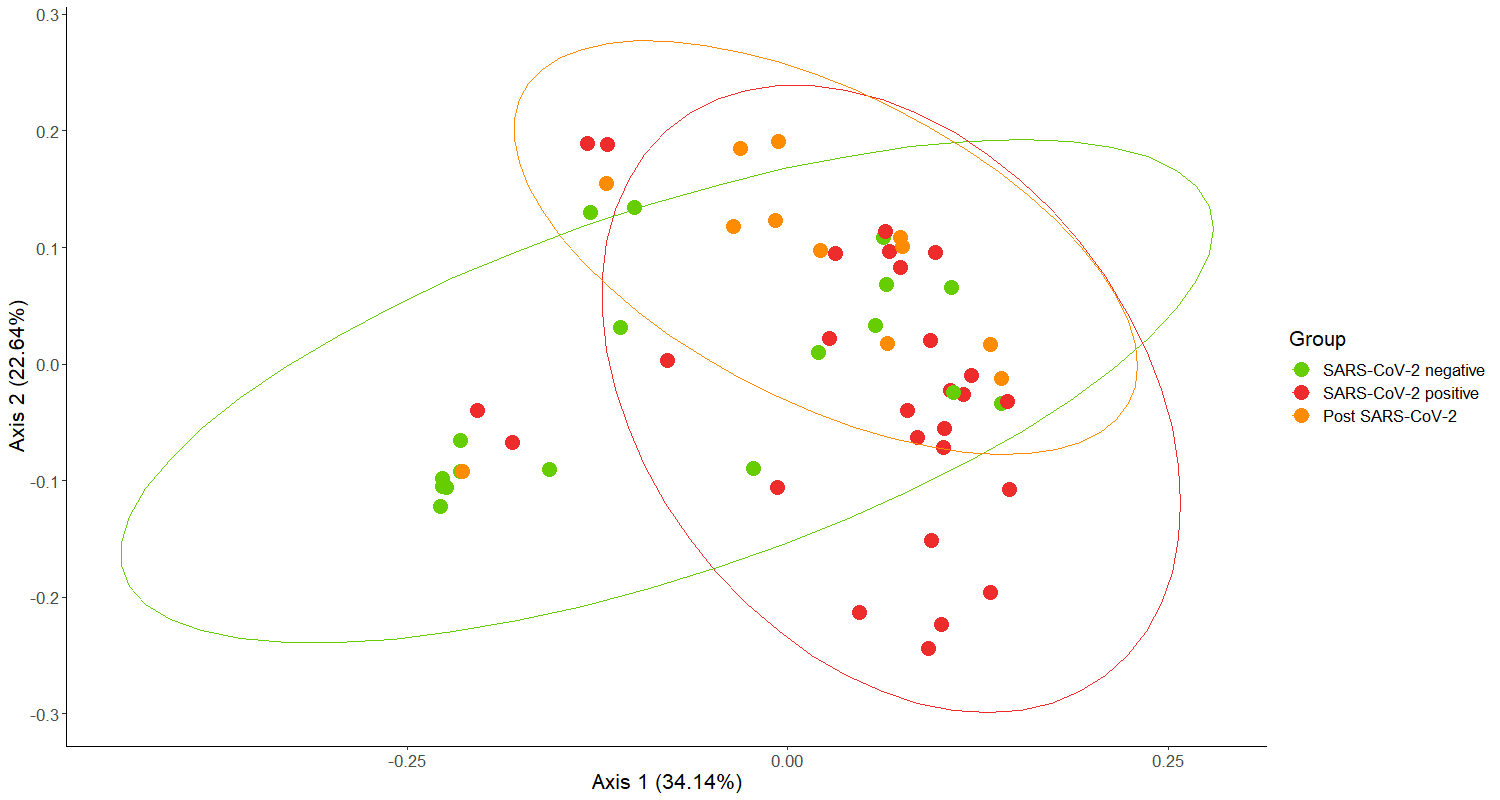

Supplement: Supplementary file 1 — Additional file 1. Principal coordinate analysis (PCoA) on weighted UniFrac distance of the gut microbiome of patient with severe acute respiratory syndrome coronavirus 2 (SARS‑CoV‑2)-related pneumonia, who were tested before and after resolution of the infection, and patients with SARS-CoV-2 unrelated pneumonia. [file 13099_2021_459_MOESM1_ESM.docx]
