# Supplementary material for: Improved gut microbiota features after the resolution of SARS‑CoV‑2 infection
Source: Gut Pathog. 2021 Oct 16;13:62. doi: 10.1186/s13099-021-00459-9 (PMC8520333; doi:10.1186/s13099-021-00459-9)
Supplement: Supplementary file 4 — Additional file 4. Supplementary methods. [file 13099_2021_459_MOESM4_ESM.docx]

**Sample manipulation, DNA extraction and library preparation**

Stool samples were self-collected, using standard protocols and regulation, in sterile containers and were stored at -80°C until processing. Microbial DNA was isolated from ≈200 mg of each homogenized fecal sample in CTAB buffer, by using DANAGENE MICROBIOME Fecal DNA kit (DanaGen-Bioted, S.L.) following manufacturer’s instruction [1]. DNA extraction was performed in a strictly controlled and sterile BSL-2 workplace and then quantified using Qubit 4.0 fluorometer (Life Technologies) and was stored at -20˚C for further study.

The V3-V4 hypervariable regions of the 16S rRNA gene were amplified in each sample by using the following primers: 5’-TCGTCGGCAGCGTCAGATGTGTATAAGAGACAGCCTACGGGNGGCWGCAG-3’ and 5’-TCTCGTGGGCTCGGAGATGTGTATAAGAGACAGGACTACHVGGGTATCTAATCC-3’ [2].

The resulting amplicons, purified by using Agencourt AMPure XP beads (Beckman Coulter), were barcoded with the Nextera XT indexes (Illumina). The obtained indexed amplicons were subsequently quantified, equimolarly diluted and then pooled to prepare a 2x250 paired ends sequencing performed on the Illumina MiSeq instrument. To increase base diversity, the internal control PhiX v3 (Illumina) was added to the final library.

**Bioinformatics Analyses**

Raw sequencing data were demultiplexed and FastQ forward and reverse reads were then analyzed by using Qiime2 (version 2020.6) [3]. FastQ reads were trimmed to remove Illumina adapters and primer sequences. Quality filtered reads were joined to obtain Amplicon Sequence Variants (ASVs) by DADA2, which provided to remove chimeras [4]. Taxonomic annotation was achieved by pre-fitted sklearn-based classifier and SILVA 132 database [5]. Final data were pre-processed removing mitochondrial sequences [6]. Statistical analysis of microbiota diversity was performed in R studio (https://www.rstudio.com/; version 4.0.2) using phyloseq package [7].

α-Diversity was evaluated based on rarefied data, using Shannon index, Inverse Simpson and Pielou’s Evenness. Statistical significance was assessed by Kruskal-Wallis test or Wilcoxon test. β-Diversity was computed by Weighted Unifrac distances at genus level. Distance matrixes were used to build a Principal coordinate analysis (PCoA) plot to demonstrate the overall dissimilarity of bacterial communities between the selected groups [8]. Significance was investigated by using Permutational multivariate analysis of variance (PERMANOVA) [9].

Differential abundances were investigated at family level using LEfSe (Linear discriminant analysis Effect Size) [10] and DESeq2 analyses [11], performed on pre-processed, not rarefied, reads, as described previously. Both analyses were carried out matching SARS-CoV-2 positive samples against SARS-CoV-2 negative samples, and SARS-CoV-2 positive samples against Post SARS-CoV-2 samples. Results are reported in the manuscript and in the Supplementary Table 1 and 2.

To investigate significance among clinical variables, Wilcoxon signed rank test and chi-square test were performed by using IBM SPSS Statistics software.

REFERENCES

1. Posteraro P, et al. Gut Pathog. 2019; 11:44.
2. KlindworthA, et al. Nucleic Acids Res 2013; e1.
3. Bolyen E, et al. Nature Biotechnology 2019; 852–857.
4. Callahan BJ, et al. Nature methods 2016; 581.
5. Pedregosa F, et al. Journal of Machine Learning Research 2011; 2825–2830.
6. Karstens L, et al. mSystems 2019.
7. McMurdiePJ, et al. PLoS One 2013; e61217.
8. Lozupone CA, et al. Appl Environ Microbiol 2007; 1576–1585.
9. Anderson MJ. Wiley StatsRef: Statistics Reference Online, 1-15
10. Segata N et al. Metagenomic biomarker discovery and explanation. Genome Biol 2011;12:R60.
11. McMurdie PJ et al. Waste not, want not: why rarefying microbiome data is inadmissible. PLoS Comput Biol 2014;10:e1003531.
